# Aspiration of a Row of Artificial Dentures in an Adult: A Case Report

**DOI:** 10.31729/jnma.7314

**Published:** 2022-04-30

**Authors:** Malati Dulal, Prashant Tripathi, Amit Shrestha, Kunjan Acharya, Sailesh Niroula

**Affiliations:** 1 Department of Ear, Nose, Throat-Head and Neck Surgery, Tribhuvan University Teaching Hospital, Maharajgunj, Kathmandu, Nepal; 2 Department of Anesthesiology, Tribhuvan University Teaching Hospital, Maharajgunj, Kathmandu, Nepal

**Keywords:** *bronchoscopy*, *case reports*, *dentures*, *foreign bodies*

## Abstract

Clinical findings of foreign body aspiration, generally, are subtle. Scrutinous clinical suspicion is always recommended. Here, we present a rare case of an adult male, who under the influence of alcohol had aspirated a row of his artificial denture without his conscience of where his denture got missing and presented to our outpatient department with non-specific symptoms. With clinical examination and advanced diagnostics, he was successfully managed with rigid bronchoscopy. With the advancement in diagnostic techniques and our widened knowledge of the condition, utmost early detection has been possible and our case report reinforces the need for a low threshold for foreign body aspiration suspicion, especially in adults with dentures, and the use of rigid bronchoscopy as a plausible tool for the prompt management of the aspiration.

## INTRODUCTION

Foreign body aspiration is incidentally uncommon in adults. Underlying impairment of protective airway mechanisms (primary neurological disorders, trauma with loss of consciousness, or sedative or alcohol use) is usually associated.^1^ Potentially life-threatening event, accounts for 0.16-0.33% of adult bronchoscopic procedures.^2^ While the majority of accidental aspiration events occur in children, adults represent up to 25% of cases.^3^ Vigilant clinical suspicion is necessary for diagnosis as clinical manifestations and roentgenograms are nonspecific in most cases. Rigid and fibre-optic bronchoscopy has become the cornerstone of successful diagnostic and even therapeutic management.^4^

## CASE REPORT

**Figure 1:**
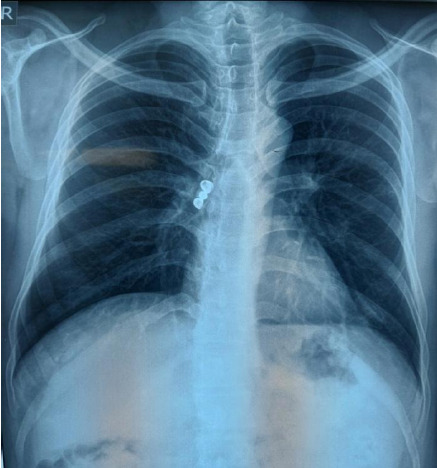
X-ray of chest showing foreign body in the right principal bronchus.

With non-specific features, a 33-year-old chronic alcohol consumer, male, presented to our Ear Nose Throat (ENT) Outpatient Department (OPD) with a history of mild productive cough for 2 weeks and with central chest discomfort while walking and sleeping for 10 days. He was referred to the Department of respiratory medicine after a chest X-ray showed a radio-opaque shadow in his right principal bronchus without signs of atelectasis (Figure 1).

Detailed history revealed that the patient had been under heavy alcohol influence alone at home, during which he suspected to have aspirated a column of three left upper molar dentures as he was unable to find them. On the 5^th^ day, he began to develop a dry non-productive cough which slowly progressed to a mildly productive cough with mucoid, non-foul smelling colourless sputum, without diurnal variation and fever. However, the cough would be aggravated while lying semi-prone to the right lateral position.

He also developed the feeling of central chest discomfort while walking a long distance and/or climbing stairs with complaints of shortness of breath (modified Medical Research Council grade 1). However, there was no history of chest pain, fever, hemoptysis, orthopnea, paroxysmal nocturnal dyspnoea, swelling of lower or upper limbs, abdomen or face, or dysphagia. The patient had no previous co-morbidities like hypertension, diabetes, obstructive airway disease, or any ENT-related illnesses. Meanwhile, he was a chronic alcohol consumer for 20 years and a chronic smoker with five pack-year consumption.

Routine blood pressure measurement showed raised the blood pressure of 170/100 mm Hg on the right arm and 160/100 mm Hg (left arm) with a regular pulse of 88 beats per minute and saturation of 95% in room air. Auscultation of the chest revealed decreased air entry on the right infra scapular region with bilateral wheeze around the infra scapular region. Results for other baseline investigations were within normal limits. Formal cardio-medicine consultation was done to work up on hypertensive urgency and an ophthalmology consultation was done to rule out insidious signs of hypertensive retinopathy. The patient was kept under intravenous empirical antibiotics. The working diagnosis of foreign body (artificial denture) aspiration was made.

**Figure 2:**
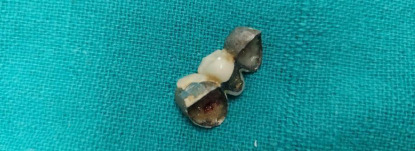
Photograph of foreign body (a row of artificial dentures) after removal.

The patient underwent emergency rigid bronchoscopy under general anaesthesia with side port ventilation in a supine position. The artificial denture row was visualised in the right primary bronchus which was removed by grasping it with forceps. Minimal granulation tissue was visualised at the site of foreign body lodgment. We performed a check bronchoscopy and with 30 minutes duration, the procedure was uneventful with no perioperative bleeding or any respiratory/airway problem (Figure 2).

Henceforth, the patient was responding to our standard conservative management and was discharged after 24 hours of post-procedural observation. Patients came for follow-up on the 3^rd^ day with a dramatic improvement in morbidity status, previously presented.

## DISCUSSION

Tracheobronchial foreign body aspiration in adults, despite being relatively rare, may present with ominous symptoms in disguise. In adults, the nature of inhaled objects is highly variable ranging from organic to inorganic material. Among inorganic materials, nail or pin aspiration occurs primarily in young or middle-aged adults during do-it-yourself activities or those with intellectual impairment or psychiatric illness. Risk factors in adults include loss of consciousness from trauma, drug or alcohol intoxication, or anaesthesia. Among organic materials; food is frequently aspirated due to incomplete chewing or poor swallowing function.^1^ Inorganic material like metal or glass items, if sharp, cause little tissue inflammation but can result in direct airway injury; which was the similar bronchoscopic finding in our case i.e. metal-reinforced artificial denture. In contrast, organic materials like nuts, beans, peas, and a variety of pills can cause significant inflammation, granulation tissue formation, and airway narrowing. In our case, minimal granulation tissue was visualised at the site of foreign body lodgment, i.e. denture metal.^5^

Compared to children, foreign body aspiration in adults usually has a subtle presentation with a paucity of image findings. Chronic cough due to distal airway obstruction is the most common symptom followed by features mimicking pneumonia i.e. fever, chest pain, hemoptysis, foul-smelling sputum, or wheeze.^4-6^ History of choking is common and dyspnea is uncommon. The most common site for aspiration in adults is the right bronchus due to its vertical angle and greater diameter than the left. As radiolucent foreign bodies are not identified on plain film, further investigation with Computed Tomography (CT) may be considered.^7^ Meanwhile, in our case the chest X-ray features were clearly indicative of denture silhouette. And with respect to our clinical history, the diagnosis led to definitive management. The additional features suggestive of foreign bodies could be posted obstructive pneumonia, atelectasis and rarely pneumothorax, and unilateral hyperinflation. In a conscious adult, the airway obstruction due to the foreign body may be relieved by chest thrust and abdominal thrust, back blows, or slaps.^8^ In case of life-threatening asphyxiation, a laryngoscopic evaluation of the oropharynx should be performed to diagnose and retrieve the supraglottic or glottic foreign body once the airway is secured. If the site of foreign body lodgment is suspected to be in central airways i.e. trachea or major bronchi, rigid or flexible bronchoscopy is generally the procedure of choice.^4^ However in our case we could successfully use rigid bronchoscopy for the extraction of a foreign body, as we located it in the right principal bronchus.

Flexible bronchoscopy is often the diagnostic procedure of choice for non-life-threatening foreign body aspiration in adults, especially for those with smaller foreign bodies in the lower airway beyond the reach of rigid instruments. Equipment like retrieval baskets, snares, forceps, grasping claws, balloon-tipped catheters, and cryoprobes is used for foreign body extraction via flexible bronchoscopes whereas rigid forceps are used for extraction via rigid bronchoscopy.^2^ Also flexible bronchoscopy provides an advantage over rigid bronchoscope, is its feasibility to be performed on moderate sedation.^4^ However, in our case, the lodgement of the artificial denture in our case was at the right principal bronchus which prompted the application of rigid bronchoscopy.

Although not validated in prospective trials, however widely practised with good outcomes, a short course (12 to 24 hours) of intravenous glucocorticoid therapy prior to extraction of foreign bodies completely encased in bulky and bleeding granulation tissue, can significantly improve the inflammatory reaction and facilitate smooth extraction.^9^ Antibiotics, as we practice in our centre, are indicated only in case of clinically, radiological, or microbiologically documented signs of respiratory tract infection.

Foreign body aspiration in adults is not common when conscious and oriented due to good protective reflexes. Although it can have a variety of airway complications, our patient had un-noticed aspiration with non-specific features and the finding of foreign body was incidental. He was successfully managed by rigid bronchoscopy and foreign body retrieval techniques.
